# Standardization of an LNA-based TaqMan assay qPCR analysis for *Aspiculuris tetraptera* DNA in mouse faeces

**DOI:** 10.1186/s12866-020-02053-6

**Published:** 2020-12-07

**Authors:** Keishiro Isayama, Kenji Watanabe, Mariko Okamoto, Tomoaki Murata, Yoichi Mizukami

**Affiliations:** 1 Institute of Laboratory Animals, Yamaguchi University Science Research Center, Yamaguchi, 755-8505 Japan; 2 Institute of Gene Research, Yamaguchi University Science Research Center, Yamaguchi, 755-8505 Japan; 3 Laboratory of Veterinary Immunology, School of Veterinary Medicine, Azabu University, Sagamihara, 252-5201 Japan

**Keywords:** Locked nucleic acid, TaqMan assay, *A. tetraptera*, Mice, PCR, Faeces

## Abstract

**Background:**

*Aspiculuris tetraptera*, as a parasitic pinworm, is most frequently detected in laboratory mice, and transmission is mediated by the eggs contained in the faeces of infected mice. A highly sensitive and quantitative faeces-based diagnostic tool would be useful for the early detection of *A. tetraptera* to inhibit the expansion of infection. In this study, we developed a quantitative assay that exhibits high sensitivity in detecting *A. tetraptera* in faeces using PCR techniques.

**Results:**

Endpoint PCR demonstrated the detection of *A. tetraptera* DNA in 0.5 ng genomic DNA extracted from the faeces of infected mice. To quantitatively detect the small amount of *A. tetraptera* DNA, locked nucleic acid (LNA)-based primers and LNA-based TaqMan probes were used for the quantitative PCR assay (qPCR). The combination of LNA-based DNA increased detection sensitivity by more than 100-fold compared to using normal oligo DNAs. The copy number of the *A. tetraptera* DNA detected was positively related to the infected faeces-derived genomic DNA with a simple linearity regression in the range of 20 pg to 15 ng of the genomic DNA. To more conveniently detect infection using faeces, the LNA-based TaqMan assay was applied to the crude fraction of the faeces without DNA purification. An assay using ethanol precipitation of the faeces yielded results consistent with those of direct microscopic observation.

**Conclusion:**

The LNA-TaqMan assay developed in this study quantitatively detects *A. tetraptera* infection in mouse faeces.

**Supplementary Information:**

The online version contains supplementary material available at 10.1186/s12866-020-02053-6.

## Background

*Aspiculuris tetraptera* and *Syphacia obvelat*a are parasitic pinworms that are most frequently detected in laboratory mice. *A. tetraptera* was found a few times in mice during routine health surveillance in our animal facilities. Infection of *A. tetraptera* with mice occurred at a frequency of 3–90% in most animal facilities, which may be affected by the breeding environment and detection system. The prevalence of *A. tetraptera* in wild mouse populations is estimated to be much higher than that in animal facilities [1]. The transmission of *A. tetraptera* to other mice is mainly mediated by eggs [2]. The laboratory mice often ingest faeces that contain pinworm eggs, and the eggs hatch into larvae that grow into adult pinworms in the proximal colon. The pinworms in the colon spawn the eggs, which are excreted together with the mouse faeces. Infection among mice is widely transmitted by the intake of faeces, including eggs in the same cage. Detection and extermination of *A. tetraptera* infection*,* in the early phase of infection is essential in laboratory animal facilities. In general, the diagnosis of *A. tetraptera* is performed by a direct detection of worms in colon contents and eggs in the faeces [3–6]. Direct observation of worms in colon contents under microscopy is the most reliable method, but animals need to be euthanized in this method. Direct observation of the eggs in faecal flotation of mice is a suitable approach with no need of euthanization during the monitor and quarantine of pathogen-infected animals; however, accurate detection of the infection is extremely difficult using a traditional faecal floatation method, as the sensitivities of traditional, non-molecular, diagnostic methods are low. As an alternative method, a PCR assay can be used to detect the genomic DNA of *A. tetraptera* [3–6]. PCR is much more sensitive than faecal floatation method, and has been shown to detect approximately 10 copies of pinworms that were undetected by faecal flotation method [6]. There has been no report indicating a quantitative PCR assay for the detection of *A. tetraptera* genomic DNA, with sensitivity as high as detecting one copy of the genome, although such a highly sensitive and quantitative assay of *A. tetraptera* detection is warranted to block transmission in the early phase of infection.

A locked nucleic acid (LNA) is an artificial nucleotide analog that is modified by a methylene bridge that connects the 2′-oxygen of the ribose moiety with the 4′-carbon; this modification reduces conformational flexibility of the nucleic acid, which results in increased binding affinity for complementary sequences and resistance to 5′-exonuclease activity [7–9]. LNA has recently been applied for the detection of the single nucleotide polymorphism [10], clinically significant mutations [11], the RNA-cleaving deoxyribozymes for the sequence-specific knockdown of mRNA [12], and in microarray platform as capture probes [13]. The LNA has also been employed to detect microorganisms, such as the hepatitis B virus [14], S*almonella* [15], unicellular parasites *Giardia* and *Cryptosporidium* [16]*,* and nematode *Meloidogyne enterolobii* [17]. As a quantitative PCR (qPCR) method, the TaqMan probe can be utilized to detect the dye of DNA, instead of using SYBR Green [18–20]. The probe used in the TaqMan assay inserts a reporter fluorescent dye into the 5′ -end of the probe and a quencher dye is linked to the 3′ -end, and the 3′ -terminal end is chemically phosphorylated to inhibit extension from the probe during PCR. The fluorescence of the intact probe is suppressed by the quencher dye due to the proximal structure of both dyes. The amplification of the target molecule by PCR leads to the cleavage of the probe by the 5′ -nuclease of DNA Taq polymerase during primer extension. Fluorescence is emitted along with degradation, and increases with the proportion of the PCR cycle number. The application of TaqMan probes is expected to improve detection accuracy of microorganisms in dirty environments and specimens, such as clinical stool samples [21]. The TaqMan probe recognizes the PCR product sequence and anneals the target DNA using both primers.

In this study, we developed a PCR assay to detect the infection of *A. tetraptera* in a qualitative and a quantitative manner using the combination of an LNA-based TaqMan probe and an LNA-based primer. The assay detects small amounts of *A. tetraptera* DNA in mouse faeces with high sensitivity of more than 100-fold compared to the previous PCR assay, and infection was also detected in crude samples without DNA purification.

## Results and discussion

### Detection of *A. tetraptera* DNA by PCR

**Fig. 1:**
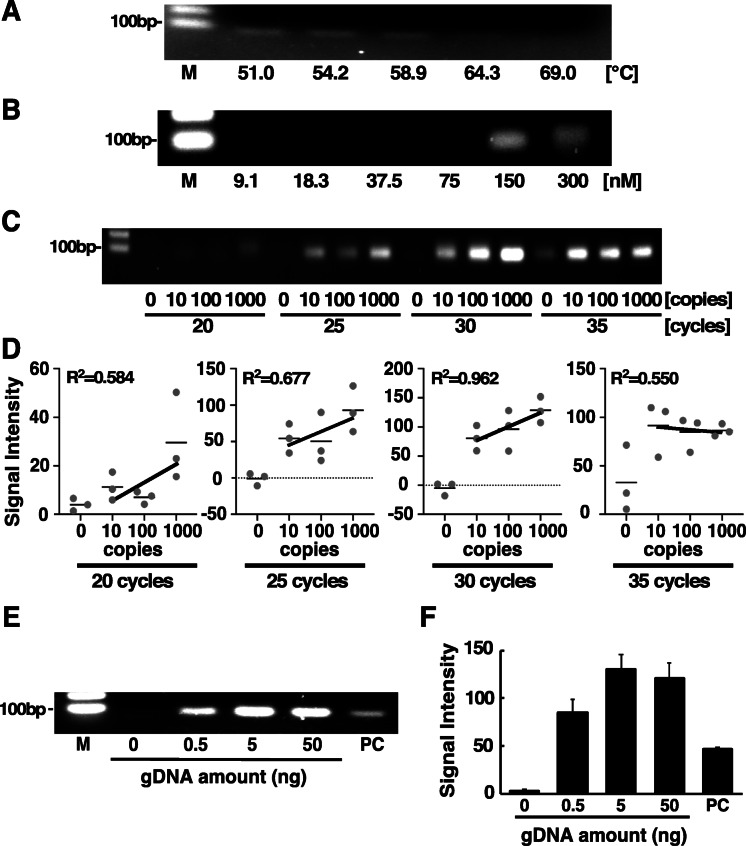
Optimizing PCR experimental conditions to detect *A. tetraptera* DNA. **a**-**c** Synthesized *A. tetraptera* DNA was amplified with Taq polymerase, as described in the “Materials and methods” section, at the indicated annealing temperature (**a**) and primer concentrations (**b**) using the indicated cycle numbers (**c**), and the products were subsequently observed by agarose gel electrophoresis using ethidium bromide staining. **d** The bands of the PCR products were quantified using the Metamorph imaging software. The signal intensity was expressed by division of total gray value by number of pixels, and corrected by background intensities. The correlation coefficient indicates the relationship between the band intensity and the copy number of synthesized *A. tetraptera* DNA in the range from 10 to 1000 copies. Data represent the mean obtained from three independent experiments. The genomic DNA were purified from the faeces of *A. tetraptera-positive* mice and amplified using the indicated amounts of genomic DNA under the optimal PCR experimental conditions. The PCR products were visualized by agarose gel electrophoresis using ethidium bromide staining (**e**), and the bands were quantified using the Metamorph imaging software (**f**). The data represent the mean ± SE obtained from three independent experiments. PC shows the data of the synthesized *A. tetraptera* DNA in the absence of genomic DNA

To detect *A. tetraptera*-derived genomic DNA in the faeces of the infected mice, we examined optimal PCR conditions using synthesized DNA corresponding to *A. tetraptera* DNA sequences. With gel electrophoresis, a PCR product of *A. tetraptera* exhibited a single band at an annealing temperature of 58.9 °C in the presence of 100 copies of the template DNA (Fig. 1a). The specific band of the PCR product was observed with a primer concentration of 150 nM with no PCR product observed at primer concentrations below 75 nM (Fig. 1b). A non-specific band at a primer concentration of 300 nM was also detected in the absence of template DNA (data not shown). The optimal number of PCR cycles was examined under these experimental conditions and the band intensities of PCR products increased with a correlation coefficient of 0.962 in a template DNA-dependent manner in the range of 10 to 1000 copies at 30 cycles (Fig. 1c and d). The band intensities were nearly constant at higher than 10 copies for 35 cycles and the co-relationship between band intensities and template DNAs was weak at 20 and 25 cycles of PCR (Fig. 1c and d). Based on these results, the endpoint PCR experiments were conducted using 30 amplification cycles with a primer concentration of 150 nM at an annealing temperature of 58.9 °C for subsequent experiments. Next, we examined whether the genomic DNA of *A. tetraptera* could be detected in the faeces derived from the infected mice under the optimized PCR experimental conditions. As shown in Fig. 1e and f, a single band was observed using at least 0.5 ng of genomic DNA extracted from the faeces of infected mice, and the product size was nearly similar to that of the positive control DNA. The DNA sequence of the PCR product was confirmed to be identical to that of *A. tetraptera* with Sanger DNA sequencing. Non-specific PCR products were unobserved in the genomic DNA. These findings show that infection of *A. tetraptera* can be detected using a small amount of genomic DNA extracted from the faeces of infected mice in a qualitative manner under PCR experimental conditions.

### Quantitative analysis of *A. tetraptera* DNA by quantitative PCR (qPCR)

**Fig. 2:**
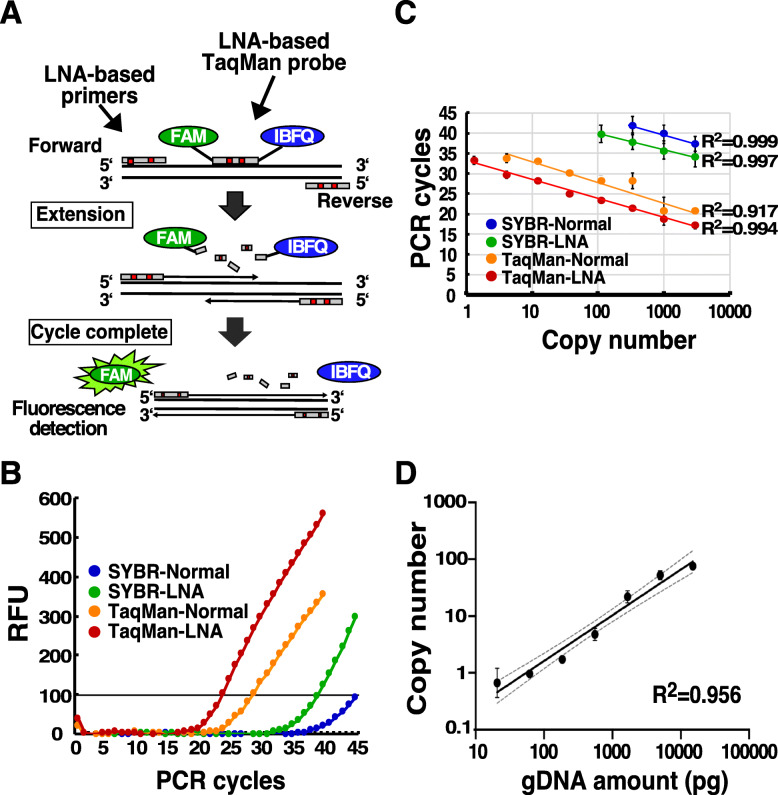
qPCR analysis of *A. tetraptera* DNA using LNA based-primers and LNA-TaqMan probe. **a** Schematic of the qPCR method using LNA-based primers and LNA-based TaqMan probe is shown. qPCR was conducted with the combination of the LNA-based primers and LNA-based TaqMan probe, which was modified with FAM, a fluorescent reporter at the 5′-end— and IBFQ, a fluorescence quencher at the 3′-end. The fluorescence of FAM within the probe disappeared by IBFQ during hybridization. The probe was cut using Taq polymerase during the extension reaction, which resulted in the emission of the reporter’s fluorescence. **b** qPCR was conducted using 100 copies of synthesized *A. tetraptera* DNA as template DNA in the presence of the indicated fluorescence reagents and primers. The detected RFU values were plotted and the representative data obtained from three independent experiments are shown. **c** qPCR was conducted using 3-fold serial dilutions of synthesized *A. tetraptera* DNA from 3000 to 1.37 copies as template DNA in the presence of the indicated fluorescence reagents and primers. The data of the copy numbers were converted to logarithm. The correlation coefficient indicates the relationship between the Ct value and the copy number of the template DNA. The data represent the mean ± SE obtained from 3 independent experiments. **d** qPCR was conducted using genomic DNA extracted from the faeces of *A. tetraptera*-infected mice in the presence of LNA-based TaqMan probes and LNA-based primers. The genomic DNA were used with a 3-fold serial dilution that ranges from 15,000 pg to 20.6 pg. The data of the copy numbers were converted to logarithm. The correlation coefficient indicates the relationship between the detected copy number of *A. tetraptera* DNA and the input genomic DNA. The dashed lines show the 95% confidence interval. The data represent the mean ± SE obtained from three independent experiments

To quantitatively analyze *A. tetraptera* genomic DNA in the faeces of infected mice with high sensitivity, we examined whether the combination of LNA-based primers and LNA-based TaqMan probes contributes to increased detection sensitivity and accuracy (Fig. 2a). As shown in Table 1, various concentrations of both primers were examined (75, 37.5, 18.8, or 9.4 nM); 9.4 and 18.8 nM were selected as optimal primer concentrations for TaqMan assay and SYBR Green assay, respectively (Supplementary Figure 1). For the annealing temperature, PCR products were equally observed at 58.9 °C with both the normal primers and the LNA-based primers (Supplementary Figure 2). Under the experimental conditions, a qPCR assay using SYBR Green reagent with oligo DNA primers demonstrated fluorescence at 40 cycles, and the intensities reached 100 RFU at 45 cycles. Substitution of the normal oligo primers with LNA-based primers revealed that the amplicons were detected at approximately 5 cycles faster compared to normal primers, which indicates that sensitivity with LNA-based primers increased by more than 30-fold compared to normal primers (Fig. 2b). The TaqMan probe also elevated detection sensitivity relative to SYBR (Fig. 2b). The combination of the LNA-based TaqMan probe and LNA-based primers revealed greater enhanced detection sensitivity of the amplicon, which was improved by more than 25 cycles compared to the SYBR assay that used normal oligo primers (Fig. 2b). The relationship between the detected copy number of DNA and the number of PCR cycles was examined for each assay. The LNA-based TaqMan assay showed that the correlation coefficient of the simple linear regression was 0.994 in the range of 1 to 3000 copies (Fig. 2c and Supplementary Figure 3). The coefficient in the LNA-based TaqMan assay was higher than in the TaqMan assay with normal primers, which resulted in improved sensitivity by more than 100-fold compared to the SYBR assay. The PCR cycle number having Ct value more than 40 at 40 cycles was defined as positive sample. The quantification limit of SYBR assay that used the normal or LNA-based primers was 3000 and 333 copies, respectively. The quantification limit of TaqMan assay that used the normal oligo primers was improved to 4.1 copies, and TaqMan assay that used the LNA-based primers was raised the quantification limit to 1.4 copies. With an LNA-based TaqMan assay, the copy number of *A. tetraptera* DNA was measured in the genomic DNA extracted from the faeces of infected mice (Fig. 2d). In the LNA-based TaqMan assay, the detected copy number of *A. tetraptera* DNA increased linearly according to the amount of genomic DNA extracted from the faeces of the infected mice (Fig. 2d). A copy of *A. tetraptera* DNA was detected in 20 pg of DNA from the faeces preparations. These findings suggest that the LNA-TaqMan assay using a combination of LNA-based TaqMan probes and LNA-based primers, with small amounts of extracted genomic DNA, can quantitatively measure the copy number of *A. tetraptera* genomic DNA in mouse faeces.

**Table 1:** Ct values in the amplification of positive control DNA with each primer concentration

Assay	Primer	Copies	Primer conc.			
			75 nM	37.5 nM	18.8 nM	9.4 nM
SYBR Green	Normal	0	36.5 ± 0.59	n.d	n.d	n.d
		100	21.6 ± 0.75	32.6 ± 1.66	43.9 ± 1.13	n.d
	LNA	0	39.3 ± 1.45	40.8 ± 0.68	44.5 ± 0.46	n.d
		100	21.9 ± 1.98	23.3 ± 0.73	36.4 ± 1.39	n.d
Taqman LNA probe	Normal	0	31.4 ± 1.57	34.8 ± 3.3	34.4 ± 0.73	42.1 ± 1.56
		100	15.7 ± 0.16	17.9 ± 1.31	18.9 ± 0.61	28.3 ± 4.19
	LNA	0	35.1 ± 3.16	30.4 ± 0.23	32.5 ± 0.52	40.2 ± 2.23
		100	17.6 ± 1.5	16.2 ± 0.23	18.3 ± 0.61	24.8 ± 2.41

Mean ± SE

### Detection of *A. tetraptera* DNA by direct PCR in faeces preparations

**Fig. 3:**
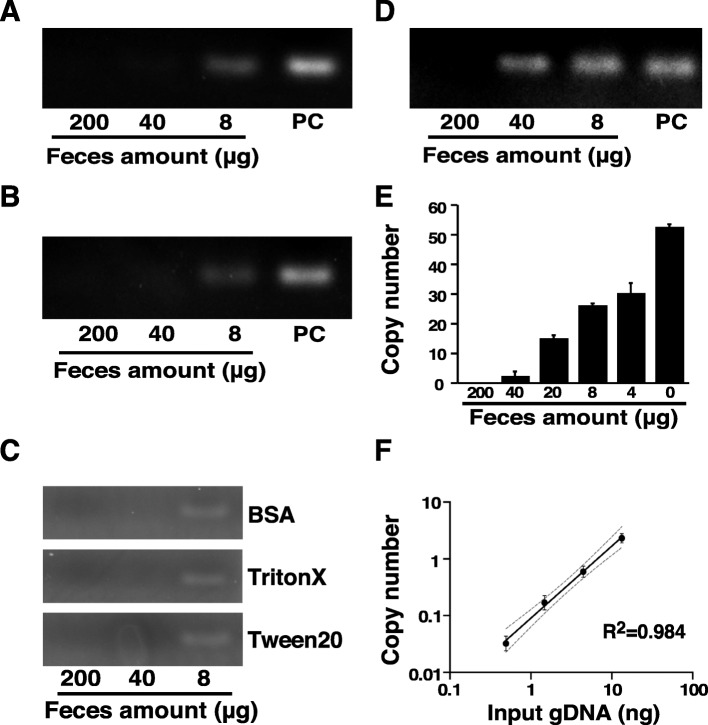
Development of direct PCR using faeces preparations in mice. **a** The indicated amounts of healthy mouse faeces were diluted with TE buffer, and the final 1 ng/μl concentration of synthesized *A. tetraptera* DNA was added to the dilutions. The DNA preparations were amplified using the optimal PCR experimental conditions, and the PCR products were observed, as described in the “Materials and methods” section. **b** The DNA preparations were treated with heat at 95 °C for 5 min after dilution, as shown in (**a**), and the PCR was performed, as described in the legend of (**a**). **c** One % BSA, 0.1% TritonX-100, or 0.1% Tween-20 was added to the DNA preparations after the dilution, as shown in (**a**), and then the PCR was performed, as described in the legend of (**a**). **d** Ethanol precipitation was conducted after the dilution as shown in (**a**), and then the PCR was performed as described in the legend of (**a**). **e** DNA preparations after ethanol precipitation were quantified using qPCR. qPCR was conducted using the LNA-based TaqMan probes and LNA-based primers as described in the “Materials and methods” section. The data represent the mean ± SE obtained from three independent experiments. **f** Four micrograms of faeces from healthy mice were added to the 3-fold serial dilutions of genomic DNA extracted from the faeces of *A. tetraptera*-infected mice (gDNA) from 13.3 ng to 0.5 ng, and subsequently precipitated by ethanol. qPCR was conducted using the LNA-based TaqMan probe and LNA-based primers as described in the “Materials and methods” section. The data of the copy numbers were converted to logarithm. The correlation coefficient indicates the relationship between the input amounts of gDNA and the detected copy numbers. The dashed lines show the 95% confidence interval. The data represent the mean ± SE obtained from three independent experiments

To conveniently detect infection of *A. tetraptera* in mice, faeces preparations were directly applied to the PCR assay without the extraction of genomic DNA. The PCR product, which corresponds to the calculated molecular size, was detected by gel electrophoresis using dilutions containing 8 μg mice faeces and the synthesized *A. tetraptera* DNA (Fig. 3a). Use of more than 40 μg of faeces resulted in the disappearance of the band, indicating that at least one component in the faeces preparations may interfere with the amplification process in the PCR step (Fig. 3a). Treatment with heat at 95 °C for 5 min did not change the dilution of the faeces, suggesting that the heat-inactivated proteins exhibit no effect on the interference of the PCR step (Fig. 3b). The addition of 1% BSA, 0.1% TrintonX-100, or 0.1% Tween-20 after heat treatment also revealed similar results to those of the dilutions (Fig. 3c). To remove any potential interference components in the mouse faeces, ethanol precipitation was implemented using faeces dilutions. In the faeces dilutions after ethanol precipitation, the band of the PCR product was observed for up to 40 μg of faeces, and the faeces amount was increased 5-fold compared to the amount with heat treatment (Fig. 3d). Pretreatment with ethanol precipitation was used to measure the copy number of *A. tetraptera* genomic DNA in mouse faeces using the LNA-TaqMan assay, which ultimately demonstrated the recovery of approximately 50% of the additional *A. tetraptera* DNA in the presence of 4 μg of faeces. The recovery amounts reduced according to the additional amount of faeces (Fig. 3e). The detected copy number correlated to additional genomic DNA in a simple linear regression with a correlation coefficient of 0.984 for genomic DNA in the range of 0.5 ng to 13.3 ng (Fig. 3f). This indicates that the LNA-TaqMan assay quantitatively detects the *A. tetraptera* genomic DNA added as a positive control in infected mouse faeces without further purification.

**Fig. 4:**
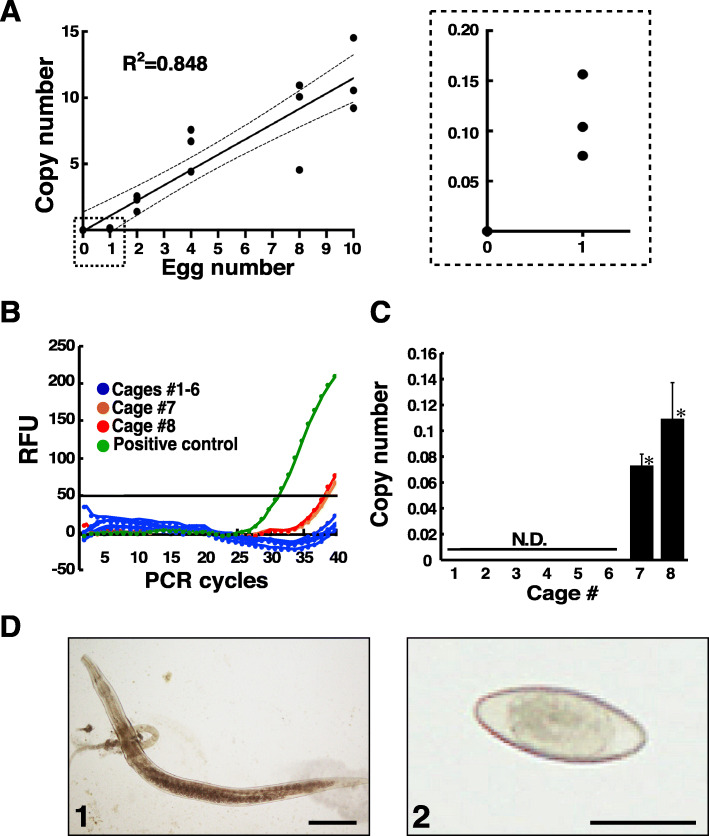
Screening of *A. tetraptera* infection in faeces of the breeding mice using the LNA-TaqMan assay. **a** The indicated *A. tetraptera* eggs were added into fresh faeces collected from healthy mice and subsequently precipitated by ethanol after the dilution with TE buffer. qPCR was conducted in triplicates using the LNA-based TaqMan probe and LNA-based primers as described in the “Materials and methods” section (left panel). The region of dashed line in the left panel was enlarged (right panel) **b** The faeces in the cages of the breeding mice were collected and diluted with TE buffer. The dilutions were precipitated by ethanol, and then qPCR was conducted using LNA-based TaqMan probes and LNA-based primers as described in the “Materials and methods” section. Representative data of the qPCR in 3 independent experiments are shown. **c** The copy number of A.tetraptera DNA in faeces collected from each cage was determined using Ct values as observed in (**b**). The data represent the mean ± SE obtained from 3 independent experiments. *P < 0.05 (one-way ANOVA with post-hocTukey’s multiple comparison test). **d** Photographs are representative data of the adult pinworm (1) in the mouse colon and the egg (2) in the faeces that were detected in the cages. Scale bars, 500 μm (1), 50 μm (2)

*A. tetraptera* eggs were collected from the faeces of the infected mice using faecal flotation method, and the relationship between egg numbers and the copy numbers using LNA-TaqMan qPCR assay was examined. The dry weight of 10 faecal pellets was 159.1 mg ± 2.8 (mean ± SE), and 3.7 eggs ±1.2 (mean ± SE) were detected in the 10 pellets (*n* = 10, Supplementary Table 1). The indicated numbers of the collected eggs were added into fresh 25 mg faeces, and the copy number of *A. tetraptera* DNA was determined by LNA-TaqMan qPCR assay with ethanol precipitation. The amplification product of *A. tetraptera* DNA was detected in the faeces including one egg, and the copy numbers were increased according to the additional egg numbers (Fig. 4a). The correlation coefficient between egg numbers and copy numbers was 0.848 (Fig. 4a). Cell division was observed in some eggs under the microscopy, causing the variation of the copy number. We examined the infection of *A. tetraptera* in the faeces of 8 cages introduced into our core facility for animal breeding using the LNA-TaqMan assay without DNA purification. The amplification of *A. tetraptera* DNA was detected in the mouse faeces of two cages (Fig. 4b), and the copy numbers were significantly increased compared to the corresponding values of the other six cages (Fig. 4c). The data in the detected copy numbers suggested that one egg was included in the used faeces. To confirm *A. tetraptera* infection in the mice of the 2 cages detected by an LNA-TaqMan assay, the contents of the colon and faeces were directly observed using a microscope. *A. tetraptera* pinworms were found in the colon (Fig. 4d1) of the infected mice, and eggs were also observed in the faeces (Fig. 4d2). No infection of *A. tetraptera* was found in the mice of the other six cages, as suggested by the PCR amplification process [22, 23].

The genomic DNA preparations might have contained interfering components derived from the faeces, based on the following reasons. First, the correlation coefficient of the simple linear regression using the genomic DNA extracted from the mice faeces was reduced as compared to that using synthesized DNA as shown in Fig. 2c and d, indicating that a component in the DNA solution may affect the assay. Second, the PCR process was nearly inhibited by large amounts of mouse faeces—amplification product was observed with dilution, which suggests interferences of the faeces in the PCR process. Lastly, among the various treatments performed, the ethanol precipitation treatment substituting the components in the dilutions was found to increase detection sensitivity, although the addition of TritonX-100 or Tween-20 inhibited secondary formation of genomic DNA in the diluted samples, as the heat treatment exhibited no effect on the PCR products. Various components, such as polysaccharides and Ca^2+^, in the faeces have been reported to inhibit the PCR process [20, 21].

It is known that one adult pinworm lays approximately 17 eggs per day, which are included in approximately 0.8 g faeces excreted in a day by a mouse [2]. In this study, approximately 22.9 eggs existed in 1 g faeces, based on the data from a traditional faecal flotation method, indicating that mouse in the cage infected with one female pinworm from the egg number including in the faeces. Eight faeces of about 200 mg were collected from each cage, and *A. tetraptera* DNA was detected with the LNA-based qPCR method in the two cages where the infected mouse was housed. These results indicated that LNA-based qPCR can detect one adult pinworm-infected mouse in the cage. *A. tetraptera* infection cannot be found by the assay using mouse faeces during 25 days after egg intake, because it takes the 23–25-day duration to develop from an egg into adult pinworm. In euthanized mice, the LNA-based qPCR could be applied to the detection of ova and nematodes cells found in gut lumen, as the components of the lumen is similar to those in faeces [24]. The detection sensitivity can be affected by the copy number of 18S rRNA in the eggs. There has been no report examining the copy number of 18S rRNA in pinworms. The copy number was estimated to be 17,440 in the egg from the dilution ratio (× 6,250) and recovery ratio (35.3%). The embryo within the egg of *A. tetraptera* in the faeces was reported to be in the morula stage having 16 cells [25], The copy number per a cell may be estimated to be approximately 1090 in the case of the morula stage. The multicopy variations ranging from 56 to 323 copies are reported to be present in nematodes [26]. The multicopy of the 18S rRNA apparently contributes to be increased the detection sensitivity, although the copy number of 18S rRNA in the eggs is accurately unclear. It would be requested the data in the time-series during the infection to compare accurately the sensitivity and quantity between LNA-based qPCR and traditional egg counts.

The LNA-based TaqMan assay developed in this study increased the sensitivity and accuracy of measurements, proving to be effective for the use of PCR assay to detect various infections, such as pinworms, in complex microbial specimens including mouse faeces.

## Conclusion

In this study, we examined the optimal conditions of qPCR to quantitatively determine *A. tetraptera* infection in mice. The band of PCR product was detected using genomic DNA extracted from the faeces of *A. tetraptera-*infected mice under optimal PCR conditions. In the quantitative detection of *A. tetraptera* DNA, the combination of the LNA-based TaqMan probe and LNA-based oligo primers improved detection sensitivity and quantitative precision. Under these PCR experimental conditions, the genomic DNA of *A. tetraptera* was detected in faeces preparations without further DNA purification.

## Materials and methods

### Materials

The primers and the TaqMan probe were designed against the unique sequence of the second internal transcribed spacer (ITS-2) in the ribosomal DNA of *A. tetraptera,* as shown in Table 2 [5]. The regions of ribosomal DNAs are widely used as primers of target PCR, because the regions are often conserved among the species and generally show low homology to other species. The primers used had less than 20% homology with the genomic DNA of *Syphacia obvelata*. The oligo DNA that was used as a positive control was synthesized for the sequence of the partial 100 bp within the ITS-2 sequences (FASMAC, Kanagawa, Japan), as follows: 5′-ATCTAAAATATACTCTTTGACGCATACACACATACACACCGTATATGT

**Table 2:** Primers and probe sequences targeting ITS-2 region of ribosome DNA in *A. tetraptera*

Primer/Probe	Sequence	Tm (°C)	Product size (bp)
Normal oligo primer Fw	ATACTCTTTGACGCATACACAC	58.2	75
Normal oligo primer Rv	CAGCCATAGGTATTGTTATATGAGC	61.4	
LNA oligo primer Fw	ATACTC**T**TTGACGCA**T**ACACAC	63	75
LNA oligo primer Rv	CAGCCATAG**G**TATTGTT**A**TATGAGC	63	
LNA TaqMan probe	FAM/CATACA**C**ACCGTAT**A**TGTGTTGC/3IABkFQ	57	–

Bold letters with underline indicates LNA-modified bases

GTTGCACATCGGCTCATATAACAATACCTATGGCTGTAGCGAGTGTTTTGTT-3′. The DNA sequence of the PCR product was confirmed with a Sanger DNA sequencer using the primers for outsides of the target sequence. A mismatch of G from A indicated by Gene Bank EF464551 was detected within the forward primer. The PCR product was identical to the target sequence, showing that the primers and the probe are useful for the measurement. The SNP except for the mismatch was undetected within the sequence of the reverse primer and the probe. Genomic DNA purified from mouse faeces that were infected with *A. tetraptera* were provided by the Central Institute for Experimental Animals as positive controls (Kawasaki, Japan). DNA LoBind tubes (Eppendorf, Hamburg, German) were used for all the experiments. Feces were obtained from C57BL/6 N mice (Japan SLC, Inc., Shizuoka, Japan). All the experiments were carried out in accordance with the recommendations in the Guide for Animal Experiments in the Yamaguchi University School of Medicine. All procedures were reviewed and approved by the Committee on the Ethics of Animal Experiments of the Yamaguchi University School of Medicine.

All other chemicals were commercially available.

### Design of LNA oligo DNAs

The LNA-inserted primers (Ajinomoto bio pharma, Osaka, Japan) and the LNA-inserted TaqMan probe (IDT, Coralville, IA) were designed according to the guidelines for building an LNA oligo product, which were suggested by EXIQON (Vedbeak, Denmark; https://www.exiqon.com/oligo-tools), as shown in Table 2. The GC-content was designed to be between 30 and 60% and to be stretches of less than 3 LNA bases. The position of the LNA residues was located approximately at the center of the primer sequence [9].

### Detection of *A. tetraptera* DNA by PCR

To determine the annealing temperature, 100 copies of the synthesized DNA (1 × 10^− 9^ ng/μl, 5 μl) were added to the 25 μl of PCR mixture containing 1x standard reaction mixture (New England Biolabs; NEB, Beverly, MA), 270 μM dNTPs (Takara Bio Inc., Shiga, Japan), 150 nM primers, and 0.17 units/μl of Taq DNA polymerase (NEB). The DNA was amplified using a PCR thermal cycler (Dicer TP600; Takara Bio) that was programmed with initial denaturation of 2 min at 94 °C, followed by 30 cycles of denaturation at 94 °C for 10 s, annealing at the temperature of 5 points in the range of 51.0 to 69 °C for 30 s, and extension at 72 °C for 30 s. The optimal annealing temperature was 58.9 °C. The optimal primer concentration was examined using the serial dilutions of 300 nM to 9.4 nM under the PCR experimental conditions described above, and 150 nM of primer concentration was determined as the optimal concentration. The cycle number of PCR was examined using 20–35 cycles in the presence of 0, 10, 100, 1000 copies using 150 nM primer concentrations at 58.9 °C annealing temperature. PCR products were amplified using 30 cycles according to copy number of genome DNA. The amplified DNA was subject to electrophoresis on 2% agarose gel and were visualized by staining with ethidium bromide for 20 min. Image scanning was performed using FluorChemFC2 (Alpha Innotech Corporation, San Leonardo, CA). The intensities of the PCR bands were measured using Metamorph imaging software (Molecular Devices, San Jose, CA). The data were obtained from three independent experiments.

### Sanger DNA sequencing

DNA sequencing was performed using a 3130xl genetic analyzer (Thermo Fisher Scientific) as described in our previous papers [27].

### Quantitative analysis of *A. tetraptera* DNA by qPCR

In a SYBR Green assay [28], 100 copies of synthesized DNA were added to a qPCR mixture containing 1x QuantiTect SYBR green buffer (Qiagen, Valencia, CA) and 18.8 nM normal oligo primers or 18.8 nM LNA-based primers in a 5-μl final volume to determine the annealing temperature. The DNA was subsequently amplified and measured using CFX384 (Bio-Rad Laboratories Hercules, CA) and programmed with initial denaturation for 15 min at 96 °C, followed by 40–45 cycles of denaturation at 94 °C for 10 s, annealing at 67.0 °C, 63.0 °C, 58.9 °C, or 56.5 °C for 30 s, and extension at 72 °C for 30 s. The PCR product was detected in both normal primers and LNA-based primers at 58.9 °C annealing temperature. To determine the primer concentrations, 100 copies of synthesized DNA or no synthesized DNA were added to a qPCR mixture containing 1x QuantiTect SYBR green buffer (Qiagen) and the serial dilutions of 300 nM to 9.4 nM of normal oligo primers or LNA-based primers, and the DNA was subsequently amplified and measured using CFX384 (Bio-Rad), programmed with initial denaturation of 15 min at 96 °C, followed by 40–45 cycles of denaturation at 94 °C for 10 s, annealing at 58.9 °C for 30 s, and extension at 72 °C for 30 s. In 18.8 nM of normal primers or LNA-based primers, the PCR product was detected in the presence of 100 copies of synthesized DNA, and was undetected in absence of synthesized DNA. In a TaqMan probe assay, 100 copies of synthesized DNA were added to the standard qPCR mixture containing 1x Sso-Advanced universal probe supermix (Bio-Rad), 100 nM TaqMan probes, and 9.4 nM normal oligo primers or 9.4 nM LNA-based primers in a 5-μl final volume to determine the annealing temperature. The DNA was subsequently amplified and measured using CFX384 (Bio-Rad) and programmed with initial denaturation for 15 min at 96 °C, followed by 40–45 cycles of denaturation at 94 °C for 10 s, annealing at 58.9 °C for 30 s, and extension at 72 °C for 30 s. One hundred copies of synthesized DNA or no synthesized DNA were added to a qPCR mixture containing 1x Sso-Advanced universal probe supermix (Bio-Rad) and the serial dilutions of 300 nM to 9.4 nM of normal oligo primers or LNA-based primers, and the DNA was subsequently amplified and measured using CFX384 (Bio-Rad) and programmed with initial denaturation for 15 min at 96 °C, followed by 40–45 cycles of denaturation at 94 °C for 10 s, annealing at 58.9 °C for 30 s, and extension at 72 °C for 30 s. In 9.4 nM of normal primers or LNA-based primers, the PCR product was detected in the presence of 100 copies of synthesized DNA, and was undetected in the absence of synthesized DNA. The threshold cycle (Ct) values of all samples were determined by setting the threshold of the relative fluorescence unit (RFU) to below 100. The calibration curve was drawn with 3-fold serially diluted synthetic oligo DNA from 3000 to 1.37 copies based on the data of three independent experiments by duplicate samples. The copy number of genomic DNA in *A. tetraptera* was calculated from the Ct value using a calibration curve. The data were determined from three independent experiments.

### Preparation of mouse faeces for direct PCR

Ten faecal pellets that were confirmed to be free of *A. tetraptera* infection by flotation method, were collected from cages that bred mice and were pooled in a conical tube. Tris-EDTA buffer at pH 8.0 (TE buffer) was added to the faeces after the pellets were mashed with a skewer. One milliliter of each sample was transferred to safe-lock tubes containing 0.3 mm glass beads and homogenized with a beads-shaker μT-12 (TAITEC, Saitama, Japan) at 3200 rpm for 30 s. The faeces were diluted to 40, 8, or 1.6 μg/μl and the suspensions used contained final faeces amounts of 200, 40, or 8 μg in PCR reactions. To determine the copy number, the faeces suspensions were diluted to 200, 40, 20, 8, 4 μg/1.5 μl. Final concentration of 100 copies synthesized *A. tetraptera* DNA (1 × 10^− 9^ ng/μl) as an internal control were included in the samples of 1 ng/μl and the samples were heated at 95 °C for 5 min. After centrifugation at 11,000×*g* for 5 min, the supernatants were collected and used directly in PCR assays. The heat treatment was conducted at 95 °C for 5 min, and afterwards, 1% BSA, 0.1% TritonX-100, or 0.1% Tween-20 were added to the dilutions. These reagents were added into the reaction solution for the following reasons. The addition of BSA leads to the improvement of PCR reaction efficiency through the association with inhibitors in the faeces [29]. The detergents such as TritonX-100 and Tween-20 result in the inhibition of the formation of DNA secondary structure and the stabilization of Taq polymerase [30]. In the ethanol precipitation process, the supernatants were precipitated at − 30 °C for 1 h with 0.1-volume of 3 M sodium acetate and 2-volume of ice cold 100% ethanol, and were subsequently centrifuged at 11,000×*g* for 15 min at 4 °C. The precipitates were washed with 70% ethanol and dried with an evaporator for 15 min. The dried precipitates were dissolved in 15 μl of TE buffer at a pH of 8.0, and DNA concentration was measured with Nano-drop (Thermo Fisher Scientific). The data were determined from three independent experiments.

### Collection of eggs from faeces of *A. tetraptera*-infected mice by faecal flotation method

Ten faecal pellets were placed in a 15 ml tube, were soaked in 1 ml saturated saline. The suspension was mashed with a skewer, and was mixed with a vortex mixer after the further addition of saturated saline. The suspension was covered with a coverslip, and was incubated for 15 min. The eggs attached to the coverslip were counted under a microscopy (BX50, Olympus, Tokyo, Japan), and were resuspended in 1 ml TE buffer. The indicated numbers of eggs were added into fresh 25 mg faeces, which were used for LNA-TaqMan qPCR assay with ethanol precipitation. The data was determined in triplicates qPCR assay.

## Supplementary Information

**Additional file 1: Supplementary Figure 1.** Optimization of primer concentration in TaqMan and SYBR methods. (A and B) One hundred copies of genomic DNA were added to a qPCR mixture containing 1x QuantiTect SYBR green buffer and the indicated concentrations of normal oligo primers (black) or LNA-based primers (red) to determine the primer concentration. The DNA was subsequently amplified and measured using CFX384 (Bio-Rad) and programmed with initial denaturation for 15 min at 96 °C, followed by 40–45 cycles of denaturation at 94 °C for 10 s, annealing at 58.9 °C for 30 s, and extension at 72 °C for 30 s. The primer concentration was determined at 18.8 nM. (C and D) One hundred copies of genome DNA were added to the standard qPCR mixture containing 1x Sso-Advanced universal probe supermix (Bio-Rad), 100 nM TaqMan probes, and the indicated concentrations of normal oligo primers (black) or LNA-based primers (red) to determine the primer concentration. The DNA was subsequently amplified and measured using CFX384 (Bio-Rad) and programmed with initial denaturation for 15 min at 96 °C, followed by 40–45 cycles of denaturation at 94 °C for 10 s, annealing at 58.9 °C for 30 s, and extension at 72 °C for 30 s. The primer concentration was determined at 9.4 nM.

**Additional file 2: Supplementary Figure 2.** Optimization of annealing temperature in SYBR method. One hundred copies of genomic DNA were added to a qPCR mixture containing 1x QuantiTect SYBR green buffer and 300 nM normal oligo primers or 300 nM LNA-based primers to determine the annealing temperature. The DNA was subsequently amplified and measured using CFX384 (Bio-Rad) and programmed with initial denaturation for 15 min at 96 °C, followed by 40–45 cycles of denaturation at 94 °C for 10 s, annealing at 67.0 °C (A), 63.0 °C (B), 58.9 °C (C), or 56.5 °C (D) for 30 s, and extension at 72 °C for 30 s. The PCR product was detected in both normal primers and LNA-based primers at 58.9 °C annealing temperature.

**Additional file 3: Supplementary Figure 3.** qPCR was conducted using 3-fold serial dilutions of synthesized *A. tetraptera* DNA from 3000 to 1.37 copies as template DNA in the presence of the SYBR and normal oligo primers (A), SYBR and LNA-based oligo primers (B), TaqMan and normal oligo primers (C), or TaqMan and LNA based oligo primers (D). The data of the copy numbers were converted to logarithm. The correlation coefficient indicates the relationship between the Ct value and the copy number of the template DNA. The dashed lines show the 95% confidence intervals. The data represent the mean ± SE obtained from 3 independent experiments.

**Additional file 4: Supplementary Figure 4.** The pictures show gel electrophoresis image of full size in Fig. 1.

**Additional file 5: Supplementary Figure 5.** The pictures show gel electrophoresis image of full size in Fig. 3.

**Additional file 6: Supplementary Table 1.** *A.tetraptera* egg number in 10 feces of mice.

**Additional file 7: Supplementary Table 2.** Tables show the raw data in the manuscript.

## Data Availability

All data generated and analyzed during this study are included in this published article and its supplementary Table 2, supplementary Figs. 4 and 5.

## Abbreviations

LNA: Locked nucleic acid
qPCR: Quantitative polymerase chain reaction
BSA: Bovine serum albumin
RFU: Relative fluorescence unit
TE: *Tris* (hydroxymethyl)aminomethane- ethylenediaminetetraacetic acid
Ct: Threshold cycle
Ca: Calcium
