# Supplementary material for: Standardization of an LNA-based TaqMan assay qPCR analysis for Aspiculuris tetraptera DNA in mouse faeces
Source: BMC Microbiol. 2020 Dec 7;20:371. doi: 10.1186/s12866-020-02053-6 (PMC7720592; doi:10.1186/s12866-020-02053-6)
Supplement: Supplementary file 1 — Additional file 1: Supplementary Figure 1. Optimization of primer concentration in TaqMan and SYBR methods. (A and B) One hundred copies of genomic DNA were added to a qPCR mixture containing 1x QuantiTect SYBR green buffer and the indicated concentrations of normal oligo primers (black) or LNA-based primers (red) to determine the primer concentration. The DNA was subsequently amplified and measured using CFX384 (Bio-Rad) and programmed with initial denaturation for 15 min at 96 °C, followed by 40–45 cycles of denaturation at 94 °C for 10 s, annealing at 58.9 °C for 30 s, and extension at 72 °C for 30 s. The primer concentration was determined at 18.8 nM. (C and D) One hundred copies of genome DNA were added to the standard qPCR mixture containing 1x Sso-Advanced universal probe supermix (Bio-Rad), 100 nM TaqMan probes, and the indicated concentrations of normal oligo primers (black) or LNA-based primers (red) to determine the primer concentration. The DNA was subsequently amplified and measured using CFX384 (Bio-Rad) and programmed with initial denaturation for 15 min at 96 °C, followed by 40–45 cycles of denaturation at 94 °C for 10 s, annealing at 58.9 °C for 30 s, and extension at 72 °C for 30 s. The primer concentration was determined at 9.4 nM. [file 12866_2020_2053_MOESM1_ESM.pdf]

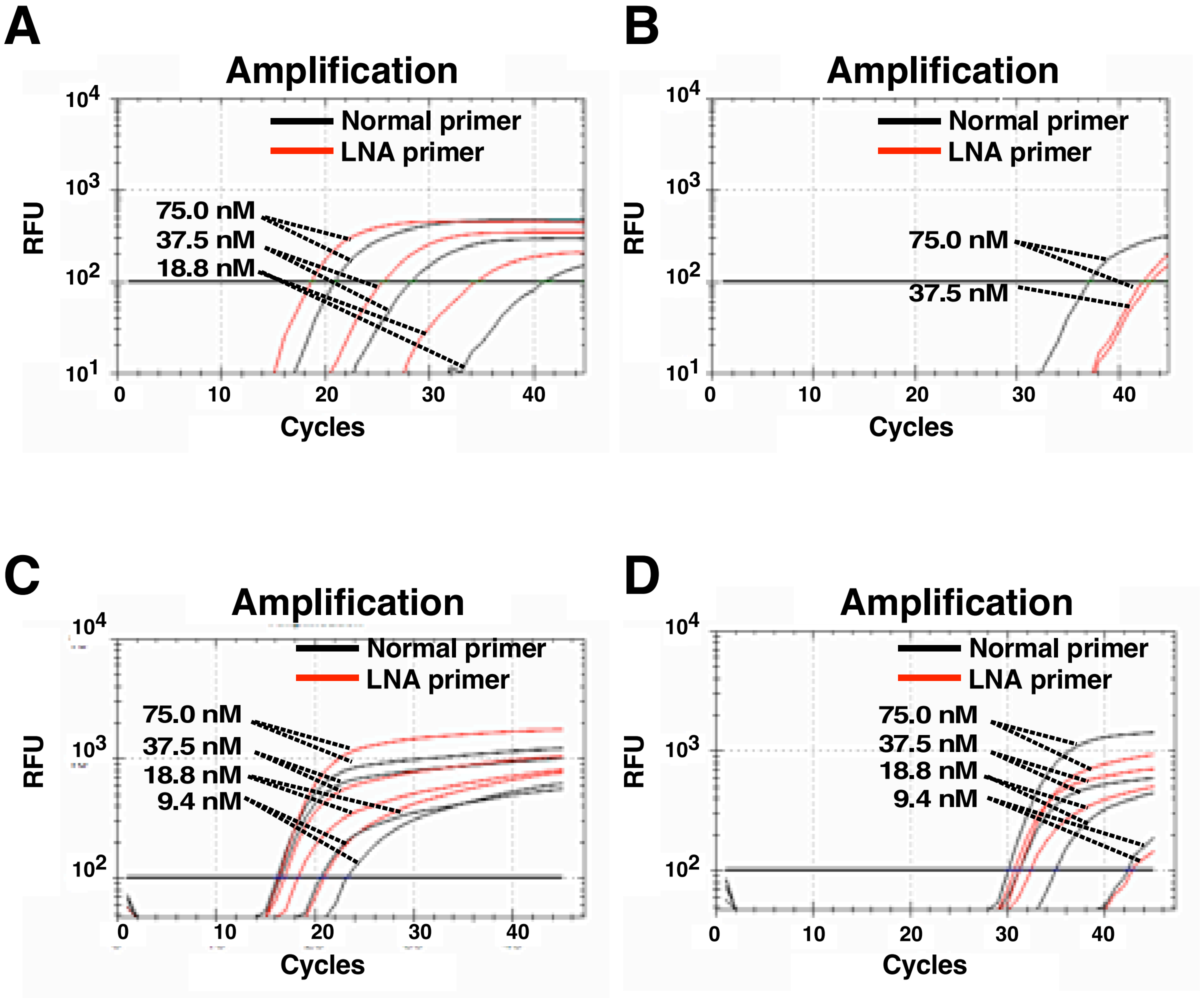

Supplementary Figure1 Optimization of primer concentration in TaqMan and SYBR methods
(A and B) One hundred copies of genomic DNA was added to a qPCR mixture containing 1x QuantiTect SYBR green buffer and the indicated concentrations of normal oligo primers (black) or LNA-based primers (red) to determine the primer concentration. The DNA was subsequently amplified and measured using CFX384 (Bio-Rad) and programmed with initial denaturation for 15 min at 96°C, followed by 40–45 cycles of denaturation at 94°C for 10 s, annealing at 58.9°C for 30 s, and extension at 72°C for 30 sec. The primer concentration was determined at 18.8 nM. (C and D) One hundred copies of genome DNA was added to the standard qPCR mixture containing 1x Sso-Advanced universal probe supermix (Bio-Rad Laboratories, Hercules, CA), 100 nM TaqMan probes, and the indicated concentrations of normal oligo primers (black) or LNA-based primers (red) to determine the primer concentration. The DNA was subsequently amplified and measured using CFX384 (Bio-Rad) and programmed with initial denaturation for 15 min at 96°C, followed by 40-45 cycles of denaturation at 94°C for 10 s, annealing at 58.9°C for 30 s, and extension at 72°C for 30 sec. The primer concentration was determined at 9.4 nM.
