# Supplementary material for: Standardization of an LNA-based TaqMan assay qPCR analysis for Aspiculuris tetraptera DNA in mouse faeces
Source: BMC Microbiol. 2020 Dec 7;20:371. doi: 10.1186/s12866-020-02053-6 (PMC7720592; doi:10.1186/s12866-020-02053-6)
Supplement: Supplementary file 2 — Additional file 2: Supplementary Figure 2. Optimization of annealing temperature in SYBR method. One hundred copies of genomic DNA were added to a qPCR mixture containing 1x QuantiTect SYBR green buffer and 300 nM normal oligo primers or 300 nM LNA-based primers to determine the annealing temperature. The DNA was subsequently amplified and measured using CFX384 (Bio-Rad) and programmed with initial denaturation for 15 min at 96 °C, followed by 40–45 cycles of denaturation at 94 °C for 10 s, annealing at 67.0 °C (A), 63.0 °C (B), 58.9 °C (C), or 56.5 °C (D) for 30 s, and extension at 72 °C for 30 s. The PCR product was detected in both normal primers and LNA-based primers at 58.9 °C annealing temperature. [file 12866_2020_2053_MOESM2_ESM.pdf]

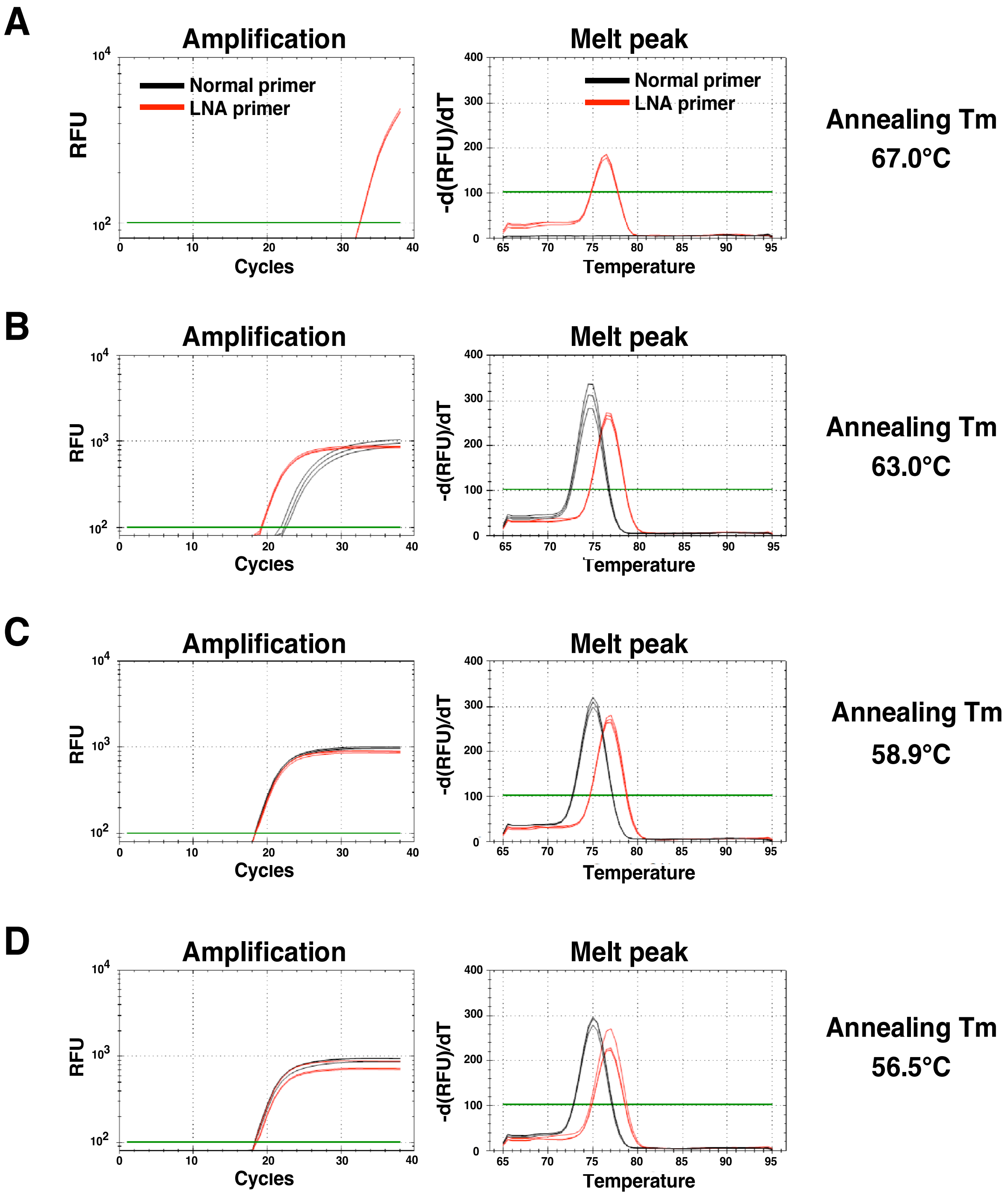

Supplementary Figure2 Optimization of annealing temperature in SYBR method
One hundred copies of genomic DNA was added to a qPCR mixture containing 1x QuantiTect SYBR green buffer and 300 nM normal oligo primers or 300 nM LNA-based primers to determine the annealing temperature. The DNA was subsequently amplified and measured using CFX384 (Bio-Rad) and programmed with initial denaturation for 15 min at 96°C, followed by 40–45 cycles of denaturation at 94°C for 10 s, annealing at 67.0°C (A), 63.0°C (B), 58.9°C (C), or 56.5°C (D) for 30 s, and extension at 72°C for 30 sec. The PCR product was detected in both normal primers and LNA-based primers at 58.9°C annealing temperature.
