# Supplementary material for: Standardization of an LNA-based TaqMan assay qPCR analysis for Aspiculuris tetraptera DNA in mouse faeces
Source: BMC Microbiol. 2020 Dec 7;20:371. doi: 10.1186/s12866-020-02053-6 (PMC7720592; doi:10.1186/s12866-020-02053-6)
Supplement: Supplementary file 3 — Additional file 3: Supplementary Figure 3. qPCR was conducted using 3-fold serial dilutions of synthesized A. tetraptera DNA from 3000 to 1.37 copies as template DNA in the presence of the SYBR and normal oligo primers (A), SYBR and LNA-based oligo primers (B), TaqMan and normal oligo primers (C), or TaqMan and LNA based oligo primers (D). The data of the copy numbers were converted to logarithm. The correlation coefficient indicates the relationship between the Ct value and the copy number of the template DNA. The dashed lines show the 95% confidence intervals. The data represent the mean ± SE obtained from 3 independent experiments. [file 12866_2020_2053_MOESM3_ESM.pdf]

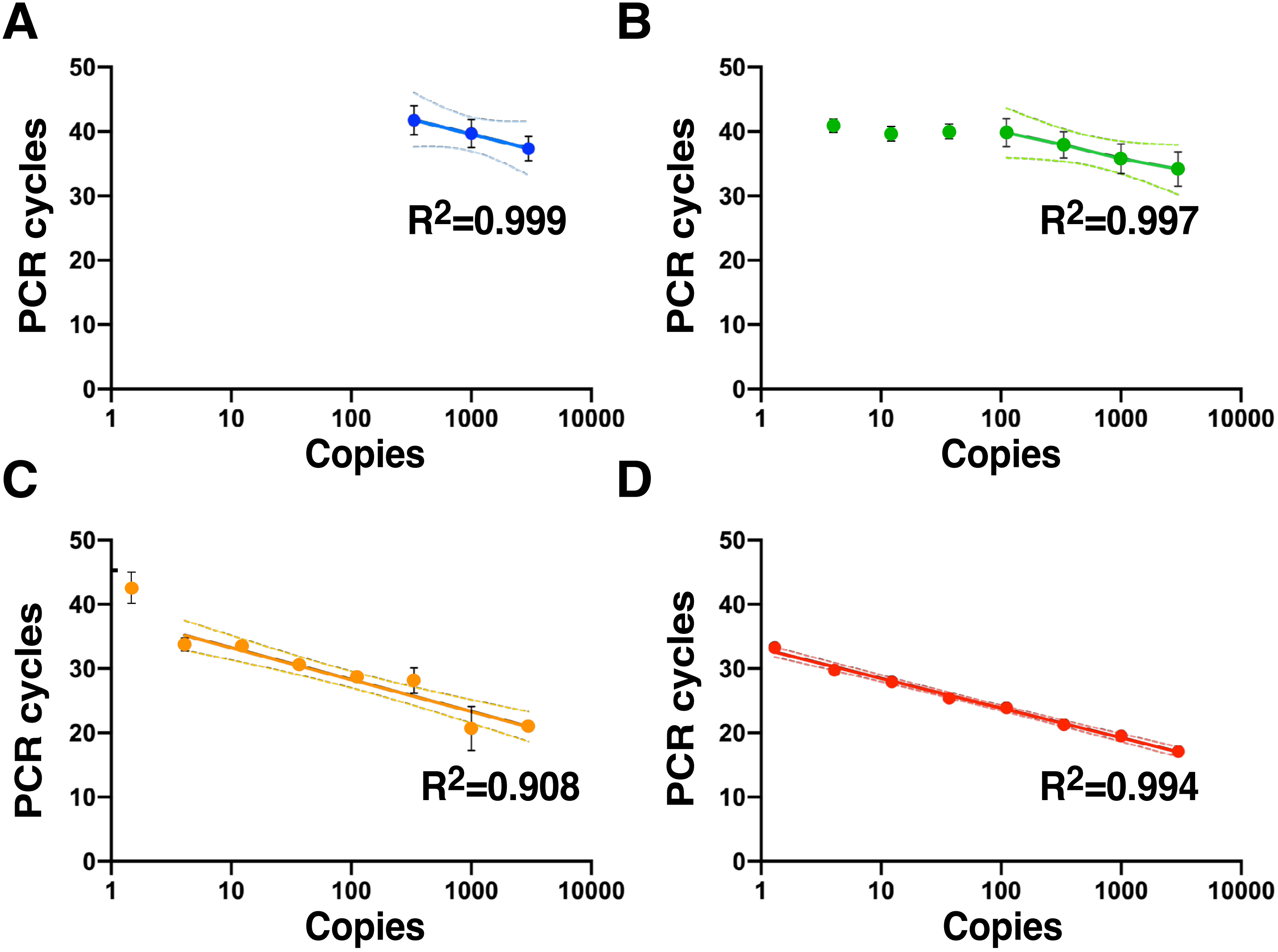

Supplementary Figure3
qPCR was conducted using 3-fold serial dilutions of synthesized *A. tetraptera* DNA from 3,000 to 1.37 copies as template DNA in the presence of the SYBR and normal oligo primers (A), SYBR and LNA-based oligo primers (B), TaqMan and normal oligo primers (C), or TaqMan and LNA based oligo primers (D). The data of the copy numbers were converted to logarithm. The correlation coefficient indicates the relationship between the Ct value and the copy number of the template DNA. The dot lines show the 95% confidence intervals. The data represent the mean ± SE obtained from 3 independent experiments.
