# Supplementary figures and images for: Standardization of an LNA-based TaqMan assay qPCR analysis for Aspiculuris tetraptera DNA in mouse faeces
Source: BMC Microbiol. 2020 Dec 7;20:371. doi: 10.1186/s12866-020-02053-6 (PMC7720592; doi:10.1186/s12866-020-02053-6)

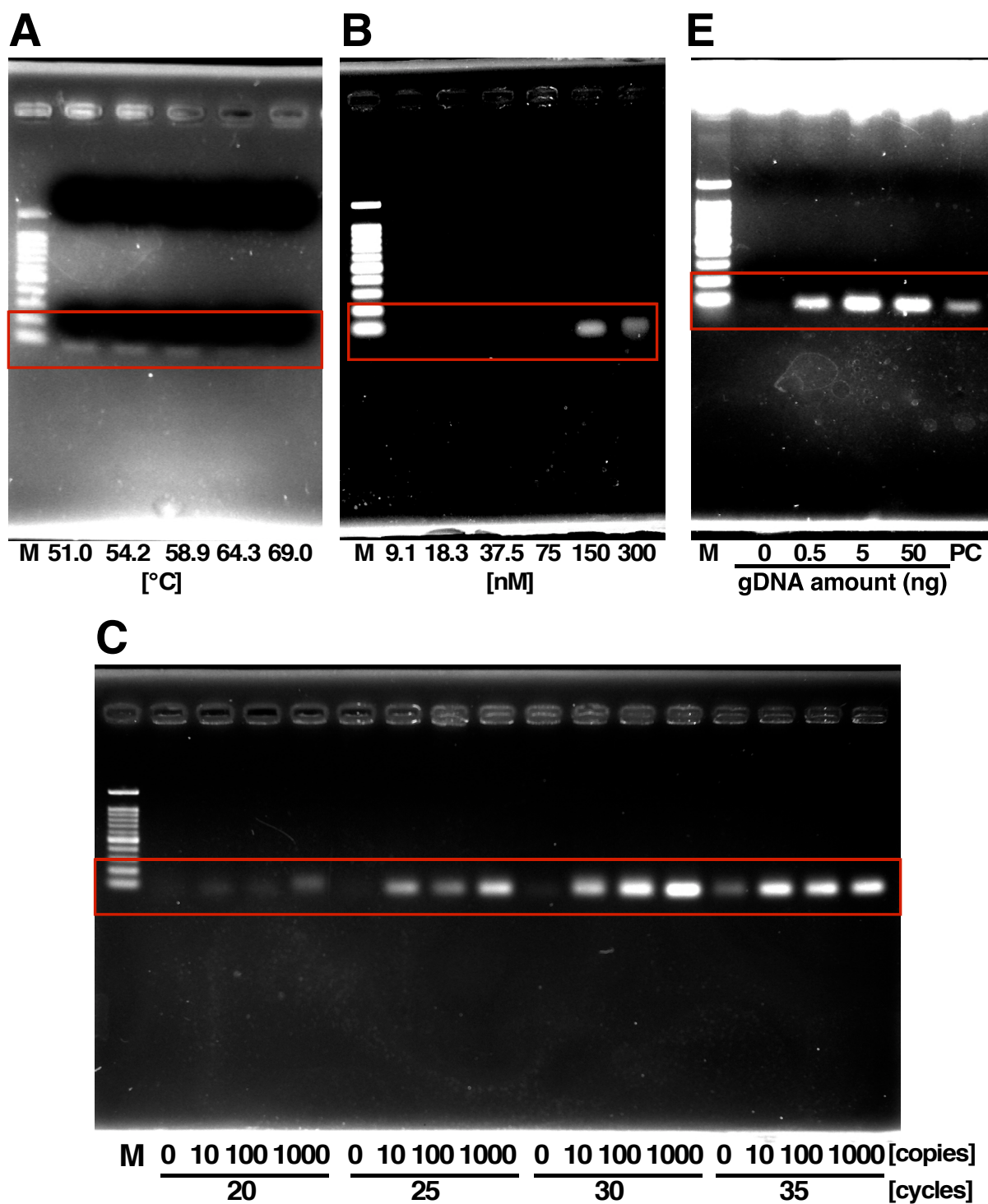

#### Supplementary Figure4

The pictures show gel electrophoresis image of full size in Fig.1

Supplement: Supplementary file 4 — Additional file 4: Supplementary Figure 4. The pictures show gel electrophoresis image of full size in Fig. 1. [file 12866_2020_2053_MOESM4_ESM.pdf]

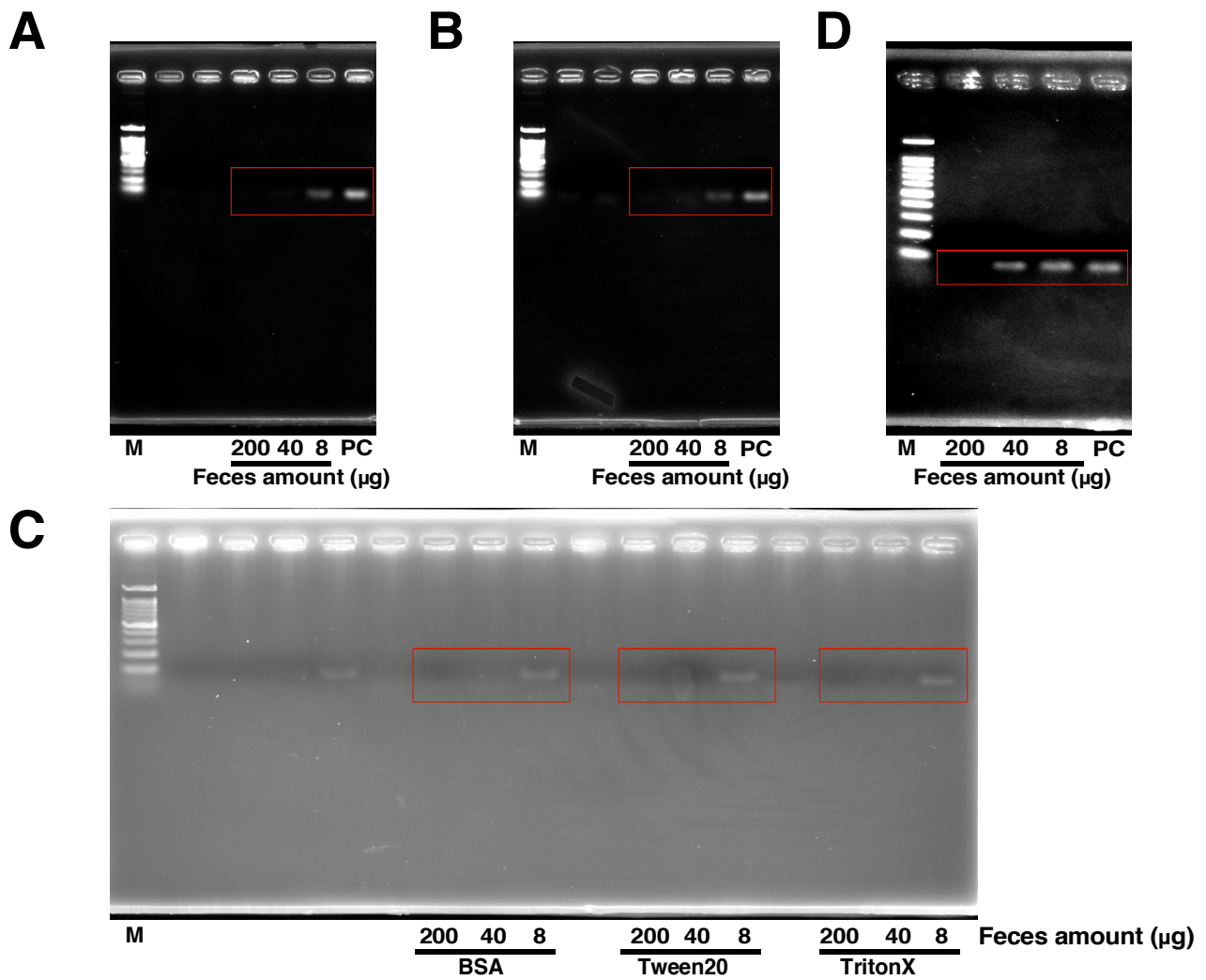

### Supplementary Figure5

The pictures show gel electrophoresis image of full size in Fig.3

Supplement: Supplementary file 5 — Additional file 5: Supplementary Figure 5. The pictures show gel electrophoresis image of full size in Fig. 3. [file 12866_2020_2053_MOESM5_ESM.pdf]
